# Validation of the Rage Attack Questionnaire-Revised (RAQ-R) in a Mixed Psychiatric Population

**DOI:** 10.3389/fpsyt.2021.724802

**Published:** 2021-08-18

**Authors:** Lisa Palm, Martina Haas, Anna Pisarenko, Ewgeni Jakubovski, Kirsten R. Müller-Vahl

**Affiliations:** Clinic of Psychiatry, Social Psychiatry and Psychotherapy, Hannover Medical School, Hanover, Germany

**Keywords:** rage attacks, RAQ-R, anger attacks, outbursts, psychiatric disorders, ADHD, personality disorder, Tourette syndrome

## Abstract

Rage Attacks (RA) represent a clinically relevant symptom in patients with different psychiatric disorders. However, only recently the Rage Attack Questionnaire Revised (RAQ-R, 22 items, range, 0–66) has been developed as a new instrument for the assessment of RA. This study aimed to validate the RAQ-R in a large mixed psychiatric and psychosomatic sample. We tested internal consistency, convergent and discriminant validity as well as factor structure. In order to further explore the relationship of RA to other psychiatric symptoms, we calculated Pearson correlations between the RAQ-R and several other self-assessments including measurements for general psychological distress, quality of life, depression, anxiety, attention deficit/hyperactivity disorder (ADHD), impulsivity, and self-regulation abilities. Most relevant predictors of RA were examined in a multiple regression with stepwise elimination. In order to assess the manifestation of RA in different psychiatric disorders, group differences between diagnostic categories and healthy controls were calculated. Additionally, psychiatric patients were compared to patients with Tourette syndrome along RAQ-R scores. Data from healthy subjects and patients with Tourette syndrome were obtained from a previous study of our group. In this study, we included 156 patients with a wide and typical spectrum of psychiatric diseases. The RAQ-R was found to have excellent internal consistency and strong construct validity in this sample (Cronbach's α = 0.97, Average Variance Extracted = 0.58). Thus, the RAQ-R was shown to be a psychometrically sound assessment of RA in patients with different psychiatric disorders. Close constructs to RA were found to be aggression and hostility (*r* = 0.68) as well as low frustration tolerance and impulse control (*r* = 0.69). Compared to healthy controls, RA were significantly more common in the psychiatric sample (*p* < 0.001). More specifically, RAQ-R scores in all diagnostic categories assessed were higher compared to controls. Highest scores and effect sizes were found in patients with ADHD and borderline personality disorder (*p* < 0.001). Our results suggest that RA are a common and relevant symptom in many psychiatric disorders. As depression and RA showed only a moderate relation, RA should be distinguished from the concept of anger attacks, which are described as a core symptom of depression.

## Introduction

Anger is a basic emotion with important functions like mobilization of physical and psychological resources (1). Dysfunctional levels of anger, however, are associated with negative outcomes such as poor evaluation by others, interpersonal conflicts, lower self-esteem, suicidal ideation, higher cardiovascular risk, and lower therapeutic success (1–5). Dysfunctional anger often causes psychological stress and impairment for both the affected persons and their environment. What demarcates functional from dysfunctional forms of anger are “frequency, reactivity, intensity, duration, and mode of expression” (1). Dysfunctional anger is therefore a clinically highly relevant symptom in many patients suffering from different psychiatric disorders [for an overview see (1, 5, 6)].

So far, research on dysfunctional outbursts of anger in psychiatric disorders focused on the concept of anger attacks, which was first introduced by Fava et al. (7, 8). According to them, anger attacks are defined as sudden outbursts of anger combined with autonomic arousal resembling panic attacks (i.e., sweating, trembling, tachycardia) (8, 9). Anger attacks are believed to represent a variant of depression, because of their high prevalence in patients with depression and the finding that treatment with antidepressants such as fluoxetine improves anger attacks (7, 8, 10, 11). Therefore, Fava et al. developed the Anger Attack Questionnaire (AAQ) to assess anger attacks in patients with depression (8). Not surprisingly, research on anger attacks in psychiatric disorders other than depression identified comorbid depression as predictor of anger attacks (12–14). As such, the concept of anger attacks and the AAQ as corresponding assessment seem inappropriate to examine dysfunctional outbursts of anger in psychiatric disorders other than depression and without comorbid depression, respectively.

Besides anger attacks, dysfunctional anger can alternatively manifest as rage attacks (RA), which are characterized by emotional control difficulties that are uncharacteristic of the person's personality and inappropriate with regard to the triggering situation (15). Originally, the concept of RA has been developed in relation to research in Tourette syndrome (TS) by Budman et al. (15). She was the first who described RA as a common and typical symptom in affected children and adolescents. Consecutively, Budman et al. developed the Rage Attack Questionnaire (RAQ), a parent questionnaire, to measure RA specifically in children with TS (15).

To overcome limitations of the RAQ (15), only recently, our group developed a revised version, the RAQ-R, a self-assessment for adults to measure different psychological and behavioral qualities/dimensions of RA (16). Therefore, we defined RA as “sudden, mostly short-lived, intensive, impulsive, emotional reactions to situations and/or stimuli that cannot be controlled” (16). Furthermore, the behavior must be totally out of proportion to the trigger event. According to our definition, rage attacks may manifest in inappropriate verbal utterances, property damage, or aggressive actions. Those affected must be aware of the disproportionate nature of their behavior. Finally, we specified that affected persons feel unable to change their behavior, although RA are perceived as unpleasant, unintentional, and often shame-filled (16). In this recent study, we examined face and content as well as construct validity of the RAQ-R in a sample of 645 healthy subjects (16). Discriminant validity was established on the basis of low to moderate correlations with a variety of psychiatric assessments of attention deficit/hyperactivity disorder (ADHD), TS, obsessive compulsive disorder (OCD), and general psychopathology. In terms of convergent validity, we employed scales on impulsivity. However, they all showed only low to moderate correlations so that convergent validity could not be established. In contrast, we were able to show good to excellent reliability and inter-item correlations. Finally, we were able to demonstrate that RA occur significantly more often in adults with TS (*n* = 127) compared to healthy controls (*n* = 645, *p* < 0.001). However, in both groups RA were significantly associated with reduced quality of life (16).

Although the concept of RA was first employed in patients with TS, we assumed that RA describe a more comprehensive form of outbursts that is not necessarily linked to a specific syndrome or disorder. In this study, we therefore aimed to (i) examine psychometric properties of the RAQ-R in a general psychiatric sample, (ii) explore differences in RA in a general psychiatric sample compared to healthy controls and to patients with TS, (iii) assess differences in RA between patients with different psychiatric diagnoses according to ICD-10, (iv) examine correlations of RA with a spectrum of other psychiatric symptoms as well as sociodemographic characteristics, and (v) identify possible predictors of RA.

## Materials and Methods

### Patients and Study Design

Based on a power calculation, we intended to recruit 120 adult patients from out-, day-, and inpatient clinics at the Departments of Psychiatry, Social Psychiatry and Psychotherapy as well as Psychosomatic and Psychotherapy at Hanover Medical School (MHH). Inclusion criteria were: (a) the presence of at least one current ICD-10 diagnosis of a mental disorder (F00–F99), (b) age ≥ 18 years, (c) proficiency in German language, and (d) written informed consent. Exclusion criteria were severe cognitive or psychological impairments, which restricted understanding or answering the questions (e.g., dementia or acute delirium or intoxication).

We did not recruit a control group (CG), but used data obtained from our previous study recruited from staff and students at MHH (16). However, we excluded 34 out of the original 645 subjects, who had reported a current psychiatric diagnosis, and thus included a CG consisting of 611 healthy controls (based on self-declaration). Data from adult patients with TS (*n* = 127) was used from that same previous study without exclusion (16). These patients were recruited from our Tourette outpatient clinic and via German advocacy groups. Data from both, CG and TS were collected via an online survey between July and October 2017.

This study has been approved by the local ethics committee at MHH (no. 7781_BO_S_2018). Informed written consent was obtained from all patients before entering the study.

### Assessments

The following assessments were performed in all patients independently of current or past diagnoses:

- RAQ-R consisting of 22 items on a four-point Likert scale ranging from 0 to 3 (0 = not at all/never, 1 = a little/sometimes, 2 = strong/frequent, and 3 = very strong/very common). The sum of all items generates the total score (range, 0–66) (16).
- Visual analog scale (VAS) for quality of life (QoL) (17) to assess life satisfaction.
- Barratt Impulsiveness Scale—Short Version (BIS-15) (18) to assess impulsivity.
- Impulsive behavior scale-8 (I-8) (19) to assess impulsivity consisting of four subscales: urgency, intention, endurance, and risk taking.
- Brief Symptom Inventory (BSI) (20): the BSI is an instrument to operationalize general psychological distress through a global severity index. In addition, nine subscales are used to screen for psychological strain in the areas of somatization, obsessive-compulsion, interpersonal sensitivity, depression, anxiety, hostility, phobic anxiety, paranoid ideation, and psychoticism.
- Beck Anxiety Inventory (BAI) (21) to measure severity of clinical anxiety.
- Beck Depression Inventory (BDI-II) (22) to assess severity of depressive symptoms.
- German self-rating scale “ADHS-Selbstbeurteilungsskala” (ADHS-SB) (23) to screen for ADHD symptoms.
- Hannover Self-Regulation Questionnaire (HSRQ) (24) to assess ego functions and the ability of self-regulation. It consists of 35 items on a six-point Likert scale and encompasses five subscales: interpersonal disturbances, frustration tolerance and impulse control, identity disturbances, affect differentiation and affect tolerance, as well as self-esteem. The total score is generated of all subscales and ranges from 0 to 25 with high scores standing for high structure levels.

Patients were asked to complete assessments without any assistance (e.g., from staff) in order to reduce possible bias due to socially desirable response behavior. Data was collected pseudonymized in paper-based form. For all assessments used—besides the RAQ-R—good to very good psychometric properties regarding reliability, validity and internal consistency have been demonstrated.

In addition, demographic data were collected including age (in years), gender (female, male, other), country of birth (Germany vs. not-Germany), and level of education (no school degree, certificate of secondary education, general certificate of secondary education, general qualification for university entrance, university degree). Current psychiatric diagnoses according to ICD-10 (multiple entries possible) and the patient status (out-, day- or inpatient) were collected from patients' records. Diagnoses were assigned by treating physicians and psychologists based on clinical interviews, previous reports, and, whenever needed, structured interviews and disease specific assessments.

### Data Analysis

All questionnaires were scanned and transformed into a digital raw data set via the survey automation software EvaSys version 7.0. Analyses were conducted in RStudio version 1.2.5033. All details of the data analysis are available as a reproducible *R* script on the Open Science Framework (doi: 10.17605/OSF.IO/73Y8P).

Due to data protection law, in our previous study performed as an online survey we were allowed to collect patients' age only in age groups. In order to present comparable demographical characteristics and being able to compare the psychiatric group (PG) to the CG and TS group, we transformed the variable “age in years” to the variable “age groups” accordingly: 1 = 18–25 years, 2 = 26–35 years, 3 = 36–45 years, 4 = 46–55 years, 5 = 56–65 years, and 6 > 65 years (16).

In case of missing values multiple imputation was used with 5 iterations (25, 26). Parameter estimates were pooled using Rubin's rule (27) if possible. Otherwise, estimates were calculated for each iteration and compared to each other.

Reliability was evaluated using Cronbach's α and composite reliability ρ_C_. Contrary to α, ρ_C_ doesn't presuppose equal loadings of all items and a 1-factor-structure.

Given a normal distribution in the PG, we calculated Pearson correlations between the RAQ-R and other scores to assess convergent and discriminant validity. Due to lack of a German questionnaire assessing the same construct, we did not expect correlations of *r* > 0.8 between the RAQ-R and any questionnaires used. Instead, we expected correlations of assessments used for convergent validity of *r* ≥ 0.5 and expected them to be higher than those used for discriminant validity. For convergent validity we used the BSI-subscale “aggression and hostility,” the HSRQ-subscales “frustration tolerance and impulse control” and “affect differentiation and tolerance,” and the impulsivity scales BIS-15 and I-8. To assess discriminant validity, we used the BAI, BDI-II, and BSI global severity index. In addition, Average Variance Extracted (AVE) was used to further assess discriminant validity (28). A Principal Component Analysis (PCA) was carried out to reevaluate the factor structure and loadings.

The PG was both in total and stratified by diagnostic categories (with *n* ≥ 4) compared to healthy subjects along RAQ-R scores. For this, non-parametric (Wilcoxon rank-sum) and parametric (independent-samples *t*) tests were used depending on the group size and the visual data distribution in q-q-plots (29). Likewise, we compared the PG to patients with TS. To identify significant demographic differences between PG and CG as potential confounders, we carried out chi-square tests and calculated Cramér's V (29). In case of differences in demographic variables, we used a multiple linear regression to check, if differences in the RAQ-R scores remain after controlling for demographic differences between the samples.

To assess differences in the RAQ-R regarding patients' sociodemographic characteristics and status (out-, day-, or inpatient), we carried out ANOVA (for ordinal variables), independent-samples *t*-tests, and Wilcoxon rank-sum tests (for dichotomous variables). Correlations between RAQ-R and age were calculated using Pearson's *r* for age as an interval variable.

To identify predictors of RA in psychiatric patients, we carried out regression models. In order to find most detailed and fitting predictors, we included all demographic variables, patient status, total scores and subscores, as well as diagnostic categories with *n* > 10 in a multiple linear regression. Through stepwise elimination and Wald test we gained a regression with predictive explanatory factors of RA (30, 31). In both multiple regression models, Gauss Markov assumptions were examined via diagnostic plots. Ordinal and dichotomous scaled variables were treated as dummies. All statistical tests were two-tailed with α = 0.05. All effect sizes were interpreted according to Cohen (32).

## Results

### Demographics and Clinical Characteristics

Between August 2018 and April 2019, 394 patients were asked for participation. Out of 291 (74%) patients, who agreed to participate, 156 completed the questionnaires (corresponding to a response rate of 54%). Half (52%) of these participants were inpatients, 33% were day-patients, and 15% were outpatients. About two third (65%) of in- and day-patients were treated at the Department of Psychiatry, Social Psychiatry and Psychotherapy, and 35% at the Department of Psychosomatic and Psychotherapy. Eighty-four percent of patients were born in Germany, while 16 % were born in other countries.

Compared to controls (*n* = 611), patients were significantly older, less formally educated and included more men (all *p* ≤ 0.001, Table 1) with largest differences in education.

**Table 1:** Demographic characteristics of the mixed psychiatric group (PG) compared to the control group (CG) and patients with Tourette syndrome (TS).

**Characteristics**		**PG**	**CG**	**TS**
*n*		156	611	127
Age, mean ± sd	Years	41.15 ± 12.84		
	Clustered in 1–6	3.06 ± 1.33	2.55 ± 1.27	2.74 ± 1.29
	V		0.166^**^	0.189
Gender, *n* (%) ^a^	Female	99 (63.5)	486 (79.5)	38 (29.9)
	Male	57 (36.5)	125 (20.5)	89 (70.1)
	V		0.148^**^	0.327^**^
Education, *n* (%)	No school degree	7 (4.5)	0	3 (2.4)
	Certificate of secondary education	28 (18.1)	7 (1.1)	16 (12.6)
	General certificate of secondary education	63 (40.6)	105 (17.2)	36 (28.3)
	General qualification for university degree	28 (18.1)	259 (42.4)	37 (29.1)
	University degree	29 (18.7)	240 (39.9)	35 (27.6)
	V		0.471^**^	0.201^*^

*PG, psychiatric group; CG, control group; TS, Tourette syndrome*.

a *“other” was indicated by none of the participants. V = Cramér's V indicating the strength of association between sample membership and demographic characteristic ranging from 0 to 1, where 0 indicates no association and 1 indicates a very strong association. P-values calculated with chi-square test*:

* *p < 0.05*,

** *p < 0.001*.

Patients with a total of 86 different psychiatric diagnoses of ICD-10 type FXX.XX were included (average number of diagnoses per patient = 2.58, range, 1–6). With regard to ICD-10 type FXX.-, 30 different diagnoses were found (average number of diagnoses per patient = 2.47, range, 1–6). To enable meaningful analyses, diagnoses were grouped into the following eight diagnostic categories:

- F11–F19: “mental and behavioral disorders due to psychoactive substance use besides alcohol”
- F20.- and F23.-: “schizophrenia and other psychotic disorders”
- F32–F34: “depression”
- F40.- and F41.-: “anxiety disorders”
- F44.-, F45.- and F48.-: “dissociative and somatoform disorders”
- F60.- and F61.-: “personality disorders” (PD), which contained

° F60.31: “borderline personality disorder” (BPD)
° F60.5-8: “cluster C PD” according to the Diagnostic and Statistical Manual of Mental Disorders (DSM-5) (33)

**Figure 1:**
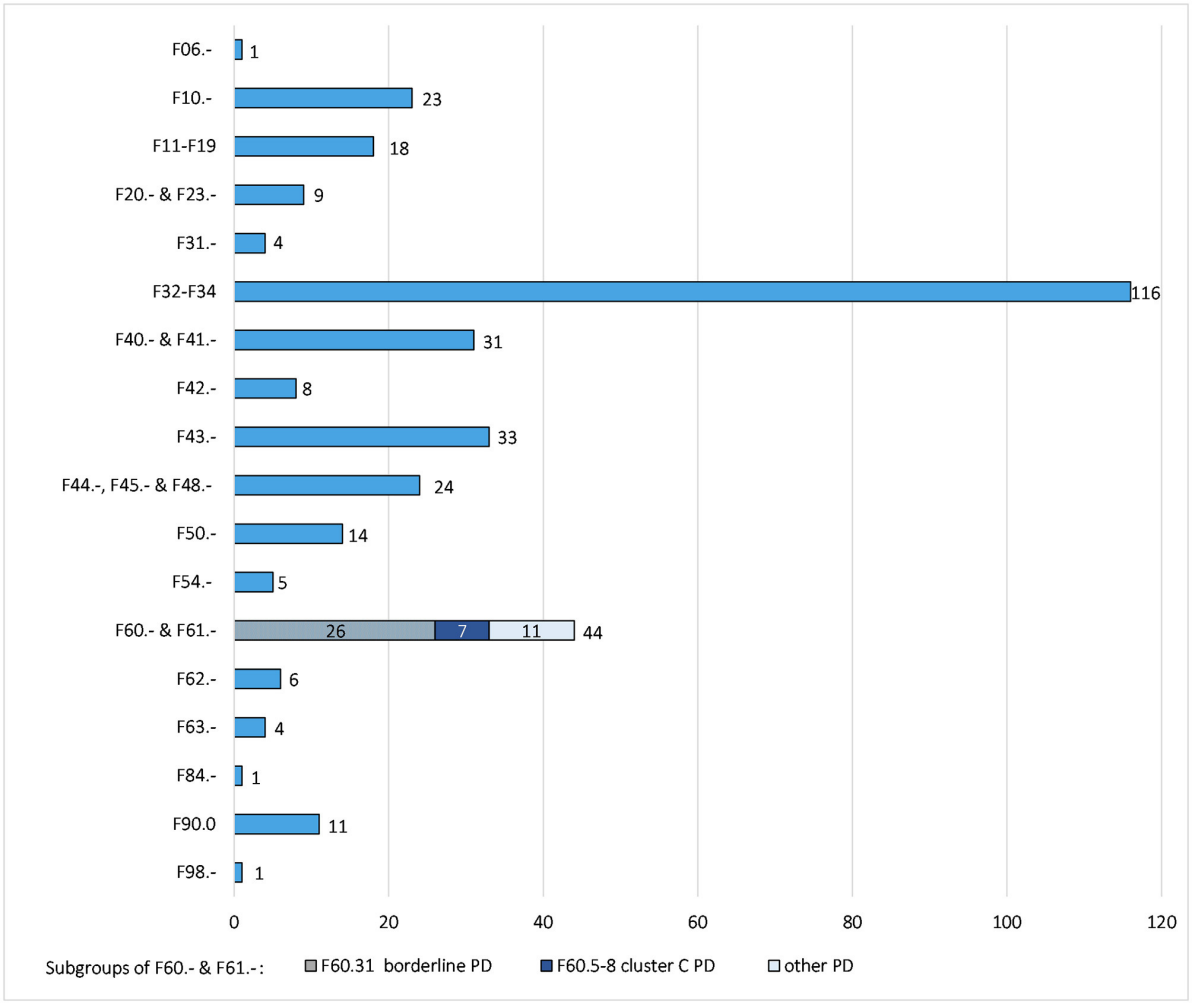
Number of different diagnostic categories in *n* = 156 patients. F06.- = other mental disorders due to brain damage and dysfunction and to physical disease, F10.- = mental and behavioral disorders due to use of alcohol, F11–F19 = mental and behavioral disorders due to psychoactive substance use besides alcohol, F20.-and F23.- = schizophrenia and other psychotic disorders, F31.- = bipolar affective disorders, F32–F34 = depression, F40.-and F41.- = anxiety disorders, F42.- = obsessive-compulsive disorders, F43.- = reaction to severe stress and adjustment disorders, F44.-, F45.-, and F48.- = dissociative and somatoform disorders, F50.- = eating disorders, F54.- psychological and behavioral factors associated with non-mental disorders, F60.-&F61.- = personality disorders, F62.- = enduring personality changes without brain damage, F63.- = habit and impulse disorders, F84.- = pervasive developmental disorders, F90.0 = attention deficit/hyperactivity disorder, F98.- = other behavioral and emotional disorders with onset usually occurring in childhood and adolescence. PD = personality disorders. The y-axis presents the ICD-10 code of the diagnosis or diagnostic category. The x-axis presents the number of patients with that diagnosis or diagnostic category. Patients with diagnoses belonging to different categories were counted for each category separately. Patients with more than one diagnosis of the same category were counted only once in that category.

Another 12 diagnoses could not be subsumed under the categories above and are thus shown as individual diagnostic categories. Thus, a total of 20 diagnostic categories is presented in Figure 1. Patients with more than one diagnosis of the same category were counted only once, while patients with diagnoses belonging to different categories were counted for each category separately.

### Missing and Ambiguous Data

In 58 patients (37.18%) at least one item was missing [from nine single questionnaires with a total of 202 items, mean per patient = 1.51 (0.75%)]. Therefore, a combined multiple imputation method was applied. All results shown contain imputed values. Two participants answered all-in-all 26 questions by crossing between two answer options. These answers were interpreted as mean values.

### Validation of the RAQ-R

We found a very high internal consistency indicated by a Cronbach's α of 0.97 and a composite reliability ρ_C_ of 0.97. A sensitivity analysis excluding patients with psychotic and bipolar disorders did not change the internal consistency. All instruments used for validation showed significant correlations with the RAQ-R (Table 2). Strong correlations were found between RAQ-R and the BSI-subscale “aggression and hostility” (*r* = 0.68) as well as the HSRQ subscales “frustration tolerance and impulse control” (*r* = 0.69) and “affect differentiation and tolerance” (*r* = 0.54) used to assess convergent validity. Weaker correlations were found with assessments used for discriminant validity (BSI global severity index: *r* = 0.43, BAI: *r* = 0.24, BDI-II: *r* = 0.32) as well as with the impulsivity scales (BIS15: *r* = 0.39, and I-8 urgency: *r* = 0.47, I-8 risk taking: *r* = 0.18, I-8 intention: *r* = −0.31, I-8 endurance: *r* = −0.32).

**Table 2:** Pearson correlations *r* between RAQ-R and other assessments.

**Assessment**	***r***	***p***
VAS-QoL	−0.170	0.027
BSI global severity index	0.426	<0.001
Aggression and hostility	0.682	<0.001
BIS15	0.390	<0.001
I-8 urgency	0.470	<0.001
Intention	−0.314	<0.001
Endurance	−0.322	<0.001
Risk taking	0.178	0.008
ADHS-SB	0.428	<0.001
BAI	0.240	<0.001
BDI-II	0.316	<0.001
HSRQ total score	0.498	<0.001
Frustration tolerance and impulse control	0.689	<0.001
Affect differentiation and tolerance	0.538	<0.001

*VAS-QoL, Visual Analog Scale for Quality of Life; BSI, Brief Symptom Inventory; BIS15, Barratt Impulsiveness Scale—Short Version; I-8, Impulsive Behavior Scale-8; ADHS-SB, Attention Deficit/Hyperactivity Disorder Self-Assessment Scale; BAI, Beck Angst Inventory; BDI-II, Beck Depressions Inventory II; HSRQ, Hannover Self-Regulation Questionnaire*.

Discriminant validity is further demonstrated by an AVE of 58% indicating that 58% of the total variance of items quantified by their factor loadings is explained by the scale.

For assessing factor structure, data was proofed suitable for a PCA with a Kaiser-Meyer-Olkin coefficient of 0.95 and *p* < 0.001 in the Bartlett test for sphericity. The scree plot confirmed the 1-factor structure, which has also been found in our previous study (16). Loadings ranged from 0.45 to 0.83 (see *R* script for details).

### Group Comparisons

**Figure 2:**
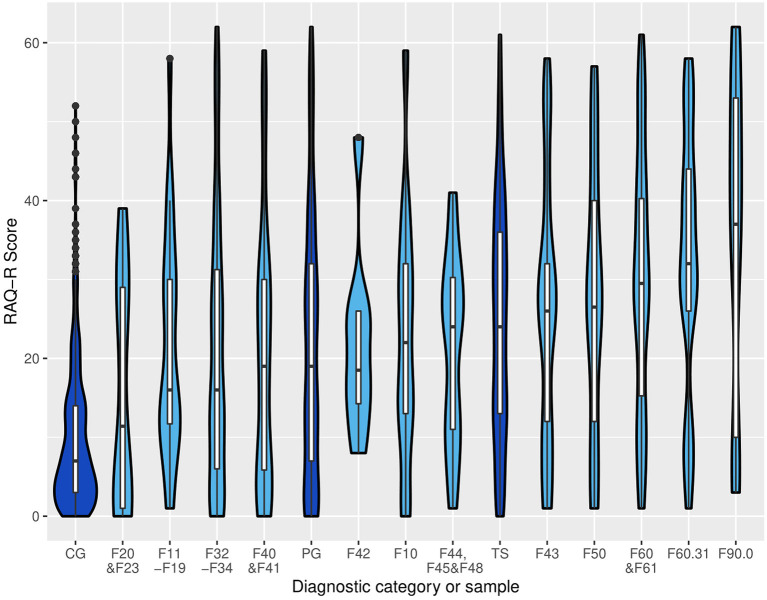
Violin plots displaying the distribution of RAQ-R scores in controls (CG), patients with Tourette syndrome (TS), mixed psychiatric group (PG), as well as diagnostic categories according to ICD-10 with *n* ≥ 8. CG = control group, F20 and F23 = schizophrenia and other psychotic disorders, F11–F19 = mental and behavioral disorders due to psychoactive substance use besides alcohol, F32–F34 = depression, F40 and F41 = anxiety disorders, PG = psychiatric group, F42 = obsessive-compulsive disorders, mental and behavioral disorders due to use of alcohol, F44, F45, and F48 = dissociative and somatoform disorders, TS, Tourette syndrome (F95.2), F43 = reaction to severe stress and adjustment disorders, F50 = eating disorders, F60 and 61 = personality disorders, F60.31 = borderline personality disorder, F90.0 = attention deficit/hyperactivity disorder. Dark blue: CG, PG, TS. Light blue: diagnostic categories within the PG. Groups and categories are sorted by median.

RA as assessed by the RAQ-R were significantly more common in the PG compared to controls with a large effect size (Table 3; Figure 2). After including age, gender, and level of education as confounders the difference between PG and CG remained highly significant with *p* < 0.001 (details of the multiple regression in the *R* script). The adjusted R^2^ for the model of 0.16 indicates a moderate goodness-of-fit.

**Table 3:** RAQ-R scores in controls (CG) compared to patients with Tourette syndrome (TS), the mixed psychiatric sample (PG), and diagnostic categories according to ICD-10 with *n* > 1.

**Sample/ diagnostic category**			***n***	**RAQ-R**			**Cohen's d**	***p* (*t*-test)**	***p* (Wilcoxo*n*-test)^a^**
				**Mean (sd)**	**Mean 95% CI**	**Median**			
CG			611	10.09 (9.33)	9.34–10.83	7.00			
TS			127	25.00 (15.36)	22.30–27.70	24.00	1.41^**^	<0.001	<0.001
PG			156	21.75 (16.93)	19.08–24.43	19.40	1.03^**^	<0.001	<0.001
	F10.-		23	23.03 (16.48)	15.91–30.16	22.00	1.34^**^	<0.001	<0.001
	F11–F19		18	21.38 (14.91)	13.96–28.79	16.00	1.19^**^	<0.001	<0.001
	F20.- and F23.-		9	15.44 (15.13)	3.81–27.07	10.80	0.57	0.092	0.608
	F31.-		4	32.50 (16.22)	6.69–58.31	32.00	2.39^*^	<0.001	0.004
	F32–F34		116	20.82 (17.14)	17.66–23.97	16.00	0.98^**^	<0.001	<0.001
	F40.-, F41.-		31	20.31 (17.04)	14.06–26.56	19.00	1.04^*^	<0.001	0.002
	F42.-		8	21.93 (12.38)	11.58–32.27	19.80	1.26^*^	<0.001	0.002
	F43.-		33	26.07 (16.71)	20.14–31.99	26.00	1.63^**^	<0.001	<0.001
	F44.-, F45.-, and F48.-		24	21.18 (11.78)	16.20–26.15	23.90	1.18^**^	<0.001	<0.001
	F50.-		14	27.27 (17.47)	17.19–37.36	26.50	1.79^**^	<0.001	<0.001
	F54.-		5	20.60 (15.68)	1.13–40.07	16.00	1.12	0.013	0.103
	F60.- and F61.-		44	29.82 (16.70)	24.74–34.90	29.50	1.98^**^	<0.001	<0.001
		F60.31	26	31.77 (16.67)	25.04–38.50	32.00	2.23^**^	<0.001	<0.001
		F60.5-8	7	27.00 (21.03)	7.55–46.45	25.00	1.78^*^	<0.001	0.014
	F62.-		6	15.67 (18.89)	(−4.15)-35.49	10.00	0.59	0.150	0.514
	F63.-		4	14.50 (15.61)	(−10.34)−39.34	14.00	0.47	0.348	0.89
	F90.0		11	35.00 (21.09)	20.84–49.16	37.00	2.59^**^	<0.001	<0.001

*CG, control group; TS, Tourette syndrome; PG, psychiatric group, F10.- = mental and behavioral disorders due to use of alcohol, F11–F19 = mental and behavioral disorders due to psychoactive substance use besides alcohol, F20.-&F23.- = schizophrenia and other psychotic disorders, F31.- = bipolar affective disorders, F32-F34 = depression, F40.-&F41.- = anxiety disorders, F42.- = obsessive-compulsive disorders, F43.- = reaction to severe stress and adjustment disorders, F44.-, F45.-, and F48.- = dissociative and somatoform disorders, F50.- = eating disorders, F54.- psychological and behavioral factors associated with non-mental disorders, F60.-and F61.- = personality disorders, F60.31 = borderline personality disorder, F60.5–8 = cluster C personality disorder, F62.- = enduring personality changes without brain damage, F63.- = habit and impulse disorders, F90.0 = attention deficit/hyperactivity disorder. Differences in RAQ-R scores compared to CG were calculated using t-test, Wilcoxon-test, and Cohen's d for effect sizes*:

* *p < 0.05 in both t-test and Wilcoxon-test*,

** *p < 0.001 in both t-test and Wilcoxon-test*.

a *According to the five imputations five p-values were calculated. The highest p-value is shown*.

Only the 17 diagnostic categories with *n* ≥ 4 were used for further group comparisons. Ten out of those contained <20 patients which limits the assumption of normal distributed data despite good visual results in the q-q-plots (see the *R* script for details). To increase reliability, we examined group differences using *t*-tests and Wilcoxon rank-sum tests for all samples and diagnostic categories (Table 3). Only for the diagnosis F54.- inconsistent results were found in terms of significance (*t*-test: *p* = 0.013, Wilcoxon-test: *p* = 0.103). Because of a very small number of patients included in this diagnosis (*n* = 5), *p*-value of the Wilcoxon-test was used for further interpretations. In all other categories, the two tests coincided in their assessment of significance (Table 3).

Compared to controls, patients of 13 out of 17 diagnostic categories demonstrated significantly higher RAQ-R scores (*p* < 0.05, in descending order of the effect sizes): ADHD, BPD, bipolar affective disorder, PD, eating disorder, cluster C PD, reaction to severe stress and adjustment disorder, mental and behavioral disorder due to use of alcohol, OCD, mental and behavioral disorder due to psychoactive substance use besides alcohol, dissociative and somatoform disorder, anxiety disorder, and depression. All these categories showed large (depression) or even very large (all others) effects. Highest mean RAQ-R scores and strongest effect sizes were found in patients with the diagnoses ADHD (F90.0), BPD (F60.31), and bipolar affective disorder (F31.-). Patients with ADHD and BPD even showed significantly higher RAQ-R scores compared to the PG without the respective disorder: ADHD: *d* = 0.86, *p* = 0.007 (*t*-test), *p* = 0.02 (Wilcoxon-test) and BPD: *d* = 0.73, *p* < 0.001 (*t*-test and Wilcoxon-test) indicating a large effect for ADHD and medium effect for BPD. For none of the diagnostic categories we found RAQ-R scores below those of the CG (Table 3).

Compared to PG [*n* = 156, mean = 21.67 (sd = 16.97), median: 19.50) patients with TS [as assessed in (16)] showed non-significantly higher mean RAQ-R scores [*n* = 127, mean = 25.00 (sd = 15.36), median = 24.00, *p* = 0.095].

Regarding sociodemographic characteristics within the PG, only age, but not gender, level of education, country of birth, and patient status showed a significantly negative correlation with RAQ-*R* (*r* = −0.20, *p* = 0.014).

### Predictors of Rage Attacks

The data set fulfilled all Gauß-Markov assumptions as shown by diagnostic plots in the *R* script. The final model of the stepwise regression showed a very high goodness-of-fit indicated by the adjusted *R*^2^ of 0.63 (Table 4). As significant predictors of rage attacks (*p* < 0.05) we identified aggression and hostility, frustration tolerance and impulse control, identity disturbances as well as the diagnostic category depression (F32–34) and the diagnosis ADHD (F90.0).

**Table 4:** Multiple linear regression using RAQ-R as dependent variable.

**Variable**		**Not-standardized coefficient**	**Standard error**	**Standardized coefficient**	***p***
Constant		2.726	2.205		0.135
BSI	Aggression and hostility	9.772^**^	1.332	0.477^**^	<0.001
HSRQ	Frustration tolerance and impulse control	7.060^**^	0.973	0.486^**^	<0.001
	Identity disturbances	−2.749^*^	0.824	−0.198^*^	0.001
ADHD (F90.0)		6.740^*^	3.373	0.398^*^	0.048
Depressive disorders (F32–F34)		−4.145^*^	1.966	−0.245^*^	0.037
R squared		0.641			
Adjusted *R* squared		0.629			
F (df = 5;150)		53.562			<0.001

*BSI, Brief Symptom Inventory; HSRQ, Hannover Self-regulation Questionnaire; ADHD, attention deficit/hyperactivity disorder*.

* *p < 0.05*,

** *p < 0.001*.

## Discussion

This study presents the first validation data of the RAQ-R in a psychiatric population. Getting back to the aims of this study as described in the introduction, the results demonstrate that the RAQ-R is a psychometrically sound assessment of RA in a mixed sample of psychiatric patients [aim (i)]. The RAQ-R shows excellent internal consistency and strong construct validity. In accordance with our previously proposed definition of RA (16), aggression and hostility, low frustration tolerance and impulse control as well as affect differentiation and tolerance were shown to be closely related constructs to RA. We were also able to demonstrate that depression, anxiety, general psychological distress, and impulsivity are rather distinct constructs. Impulsivity (as assessed by I-8 and BIS15) and RA (as assessed by RAQ-R) were shown to be less related than expected, which is in line with results from our previous study (16). All other validation tests met our expectations and indicate very good convergent and discriminant validity. Our data corroborate the 1-factor structure of the RAQ-R (16).

Our data clearly indicate that RA as assessed by the RAQ-R are more common in a mixed psychiatric sample compared to healthy subjects [aim (ii)]. Though the difference showed a very large effect size, the presence of a psychiatric diagnosis and demographic variables explained only 16% of variations of the RAQ-R scores. This finding as well as the high variance of RA in the psychiatric sample suggest strong influence by additional factors. Accordingly, we identified the presence of “aggression and hostility” (as assessed by BSI), “frustration tolerance and impulse control” (as assessed by HSRQ) as well as the diagnosis of ADHD as strong positive predictors of RA [aims (iv) and (v)]. These findings are in line with our definition of RA (16), since the BSI subscale “aggression and hostility” measures anger, irritability, rage, aggression and hostility (20) and the HSRQ subscale “frustration tolerance and impulse control” assesses impatience and the incomplete control of aggressive impulses (24).

Our results further corroborate that RA should be distinguished from the concept of anger attacks as defined by Fava et al. (8), although both describe phenomena of explosive outbursts in combination with emotional control difficulties. Anger attacks have consistently been found to be closely related to depression (9–11, 13, 34, 35). Interestingly, even in mental disorders other than depression, comorbid depression has been identified as a predictor of anger attacks (9, 12–14). In contrast, we found only a moderate correlation between RA (as assessed by RAQ-R) and depression symptoms [as assessed by BDI-II, aim (iv)]. In addition, the effect size for depression (F32–F34) was smaller compared to all other diagnostic categories [aim (iii)]. Finally, the diagnosis of depression was even identified as a *negative* predictor of RAs when controlling for other diagnoses, symptoms, and sociodemographic characteristics [aim (v)]. Thus, based on available data, RA have to be classified as a distinct symptom, while anger attacks seem to represent a core symptom of depression.

The general relevance of RA in psychiatric patients is indicated by very large effect sizes in most and higher mean RAQ-R scores in all diagnostic categories compared to healthy controls [aim (iii)]. Significantly higher RAQ-R scores compared to CG were found for the following diagnostic categories, respectively (in descending order of effect size): ADHD (F90.0), BPD (F60.31), bipolar affective disorder (F31.-), PD (F60.-and F61.-), eating disorder (F50.-), cluster C PD (F60.5-8), reaction to severe stress and adjustment disorder, TS, mental and behavioral disorder due to use of alcohol (F10.-), OCD (F42.-), mental and behavioral disorder due to psychoactive substance use besides alcohol (F11–F19), dissociative and somatoform disorder (F44.-,F45.-&F48.-), anxiety disorder (F40.- and F41.-), and depression (F32–F34). Since sample sizes were in part very small and consisted of <10 patients (as shown in Figure 1 and Table 3), caution is needed in interpreting results. Noteworthy, and in line with clinical experience and diagnostic criteria (1, 5, 33), our results suggest a high clinical relevance of RA in patients with ADHD and BPD indicated by very large effect sizes compared to healthy controls and significant differences compared to all other psychiatric patients.

Regarding demographic differences, our psychiatric sample showed significantly fewer RA with increasing age, but no significant relation between RA and gender, level of education or country of birth [aim (iv)].

In our previous study, we were able to demonstrate that patients with TS suffer from more severe RA compared to controls as assessed by RAQ-R (16). Interestingly, in TS mean RAQ-R scores were even non-significantly higher than in the mixed psychiatric population. One might argue that increased rates of RA might be influenced by comorbid ADHD, since ADHD is a common comorbidity in TS and we found highest RAQ-R scores in patients with (pure) ADHD. However, we were able to demonstrate that RAQ-R scores are also increased in “TS only” without comorbid ADHD or any other psychiatric disorder (16). Accordingly, and in line with other studies (36), RA seem to represent a common and discrete symptom in TS. Our data, therefore, further supports the view that former ICD-10 classification of TS in the category “behavioral and emotional disorders with onset usually occurring in childhood and adolescence” was much more accurate compared to the new ICD-11 classification of TS in the category of “movement disorders” (37, 38).

This study has several significant strengths. We assessed a wide spectrum of common psychiatric symptoms allowing to also analyze correlations to several close and distinct constructs. Different from most recent studies investigating clinical aspects of anger and rage attacks, we were able to compare RA in a psychiatric sample not only with healthy controls, but also between different diagnostic categories, respectively, according to ICD-10. External validity can be regarded as high for a mixed psychiatric population in a university clinic, since (a) patients from two different departments were included, (b) patients from out-, day- and inpatient clinics were included, (c) no relevant exclusion criteria have been predefined, (d) we included patients suffering from a broad and typical spectrum of different—and in many cases more than one—psychiatric diagnoses seen in psychiatric clinics, and (e) mildly to very severely affected patients were included. Another strength of our study is the availability of all raw data and analysis as reproducible *R* scripts aiming to increase the reliability and objectivity of the data processing.

The following limitations have to be taken into consideration: (a) although we included more than 150 patients, the sample size was too small to carry out a confirmatory factor analysis including an analysis of measurement invariance between groups. Nevertheless, the results of the PCA confirmed the 1-factor structure and loadings (16); (b) we used only self-assessments, but no examiner assessments; (c) for some diagnostic categories no correlations with the RAQ-R could be calculated due to small sample sizes; (d) a small number of data was missing. However, this was addressed by using multiple imputation, the gold standard approach to handle missing data (39); (e) we did not recruit a control group, but instead used data obtained from our previous study (16). Thus, an influence from different methods (paper-based vs. online survey) and time periods of data collection (7-10/2017 vs. 8/2018-4/2019) cannot completely be excluded; (f) sociodemographic characteristics between the control and patient groups slightly differed. Therefore, confounding cannot be completely ruled out, although we controlled for sociodemographic variables; (g) since no validated German version of the AAQ is available, this questionnaire could not be included in our study; (h) our validation only involves the original German version of the RAQ-R. An English version will be published in the near future, but has not been validated yet.

In conclusion, we validated the RAQ-R, a recently developed new instrument for the assessment of RA in patients with a wide spectrum of different psychiatric disorders, and found good to excellent psychometric properties. In contrast to previous assessments measuring anger or rage attacks, the RAQ-R measures the severity of RA in a dimensional way and, additionally, assesses psychological and behavioral characteristics of RA. In contrast to the AAQ (developed for the assessment of anger attacks) and the RAQ (a parents' assessment of RA only in children with TS), the RAQ-R is applicable to adult patients with the whole spectrum of psychiatric disorders. Our data provides additional support for the clinical relevance of RA in psychiatric populations, since RA were found to be a common symptom in different psychiatric disorders, but in particular in patients with ADHD and BPD.

## Data Availability Statement

The datasets presented in this study can be found in online repositories. The names of the repository/repositories and accession number(s) can be found at: Open Science Framework (doi: 10.17605/OSF.IO/73Y8P).

## Ethics Statement

The studies involving human participants were reviewed and approved by the Ethics Committee at Hannover Medical School. The patients/participants provided their written informed consent to participate in this study.

## Author Contributions

LP, KM-V, and EJ contributed to the conception and design of the study, contributed to the acquisition of data, and organized the database. LP wrote the first draft of the manuscript. LP, MH, and EJ contributed to the analysis and interpretation of data. All authors contributed to manuscript revision, read, and approved the submitted version.

## Conflict of Interest

In the context of other research, KM-V has received financial or material research support from EU (FP7-HEALTH-2011 No. 278367, FP7-PEOPLE-2012-ITN No. 316978) DFG: GZ MU 1527/3-1 and GZ MU 1527/3-2, BMBF: 01KG1421, National Institute of Mental Health (NIMH), Tourette Gesellschaft Deutschland e.V. Else-Kröner-Fresenius-Stiftung, GW pharmaceuticals, Almirall Hermal GmbH, Abide Therapeutics, and Therapix Biosiences. She has received consultant's honoraria from Abide Therapeutics, Boehringer Ingelheim International GmbH, Bionorica Ethics GmbH, CannaMedical Pharma GmbH, Canopy Grouth, Columbia Care, CTC Communications Corp., Demecan, Eurox Deutschland GmbH, Global Praxis Group Limited, IMC Germany, Lundbeck, Sanity Group, Stadapharm GmbH, Synendos Therapeutics AG, and Tilray. She has received speaker's fees from Aphria Deutschland GmbH, Almirall, Cogitando GmbH, Emalex, Eurox Deutschland GmbH, Ever pharma GmbH, Meinhardt Congress GmbH, PR Berater, Spectrum Therapeutics GmbH, Takeda GmbH, Tilray, Wayland Group. She has received royalties from Deutsches Ärzteblatt, Der Neurologie und Psychiater, Elsevier, Medizinisch Wissenschaftliche Verlagsgesellschaft Berlin, and Kohlhammer. The remaining authors declare that the research was conducted in the absence of any commercial or financial relationships that could be construed as a potential conflict of interest.

## Publisher's Note

All claims expressed in this article are solely those of the authors and do not necessarily represent those of their affiliated organizations, or those of the publisher, the editors and the reviewers. Any product that may be evaluated in this article, or claim that may be made by its manufacturer, is not guaranteed or endorsed by the publisher.
